# The ecology and geography of temnospondyl recovery after the Permian–Triassic mass extinction

**DOI:** 10.1098/rsos.241200

**Published:** 2025-03-05

**Authors:** Aamir Mehmood, Suresh A. Singh, Armin Elsler, Michael J. Benton

**Affiliations:** ^1^ School of Biological Sciences, University of Bristol, Life Sciences Building, Tyndall Avenue, Bristol BS8 1TQ, UK; ^2^ School of Earth Sciences, University of Bristol, Life Sciences Building, Tyndall Avenue, Bristol BS8 1TQ, UK

**Keywords:** geometric morphometrics, ecological function, temnsopondyli, Triassic, Permian–Triassic mass extinction

## Abstract

One of the mysteries of the Permian–Triassic mass extinction was the subsequent success of temnospondyls. Temnospondyls were key early tetrapods in the Carboniferous and Permian and hardly seem to be ideal pioneers in a tough post-extinction world. Did they survive because of some unusual adaptations or by occupying some limited part of the world? We explore temnospondyl success in the Triassic by comparing their functional ecomorphology and palaeogeographic distributions. We find that Early Triassic temnospondyls exhibited all skull sizes and shapes, reflecting a wide diversity of feeding modes: abundant parabolic-snouted forms, and less common longirostrine (long-snouted) and insectivorous (short-skulled) forms. In fact, morphospace occupation by temnospondyls increased dramatically from Late Permian to Early Triassic, and then decreased in the Middle Triassic, but without emphasis on one feeding mode or another. Nor is there any evidence for unusual patterns of evolution: Temnospondyli and subclade Trematosauria follow an Ornstein–Uhlenbeck evolutionary model, suggesting evolution towards a common skull shape. Metoposauroidea, Brachyopoidea and basal Stereospondyli evolved by the stasis model. Further, these Early Triassic temnospondyls did not occupy a limited part of the world; they show temperate distributions, but with some specimens in equatorial regions, contradicting the idea of a permanently impermeable tropical dead zone.

## 1. Introduction

**Figure 1.**
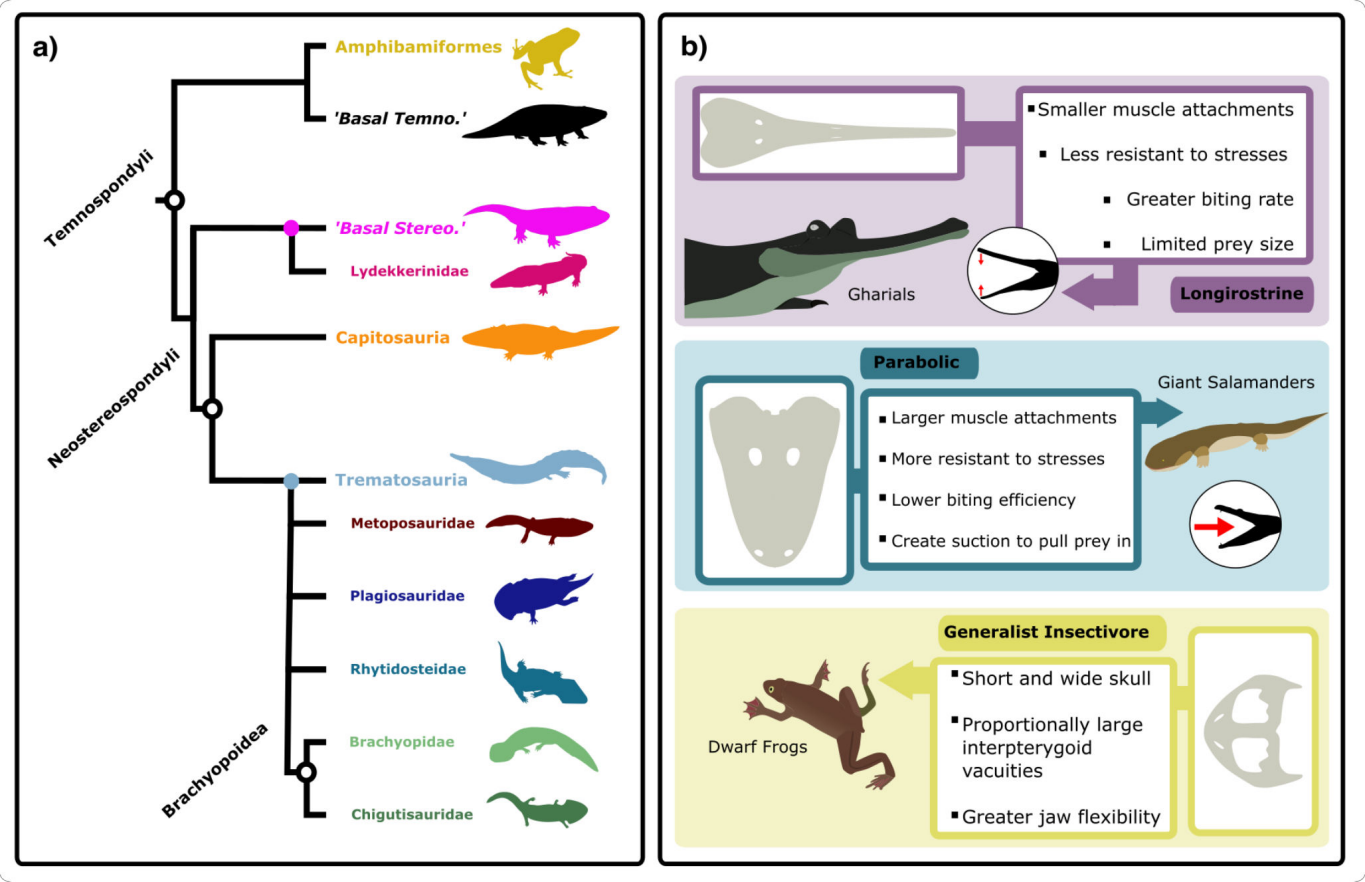
Temnospondyls of the Triassic. (a) simplified phylogeny of the temnospondyl clades investigated here, with two paraphyletic assemblages, ’basal temnos’ and basal ’stereos’. Amphibamiformes are a clade within Dissorophoidea, comprising Micropholidae and Lissamphibia. Branches stemming from coloured nodes depict subclades of this node. (b) three functional groupings based on skull morphology: large parabolic skulls characteristic of generalists such as extant crocodilians; generalist insectivore with short and wide skulls as in modern frogs; and longirostrine, adapted for fast, weak bites usually in fish eating, as in modern gharials. Abbreviations: Stereo., Sterospondyli; Temno., Temnospondyli. Silhouette images from Phylopic (https://www.phylopic.org: Dmitry Bogdanov (*Metoposaurus*, *Lydekkerina*, *Batrachosuchus*, *Pelorocephalus*, *Trematosaurus*) all CC0 3.0; Steven Traver (*Dendrobates*) CC0 1.0). Nix Draws Stuff/Nix illustration (*Eryops*) CC0 4.0. Silhouette images from Wikimedia Commons (https://commons.wikimedia.org: Smokeybjb (*Eolydekkerina magna*, *Deltasaurus kimberleyensis*); Nobu Tamura (*Gerrothorax* BW and *Wetlugasaurus* BW) all CC0 3.0). Vector graphics: Mike Prince (Gharial) CC0 2.0; Momotarou2012 (*Andrias japonicus*) CC0 3.0; CDC (African Dwarf Frog) Public Domain. Skull Graphics vectorized by AM; *Aphaneramma* [9]; *Cyclotosaurus* [1] and *Triadobatrachus* [10].

The temnospondyls were a clade of some 300 species that existed from the Carboniferous to Cretaceous (350–120 Ma, million years ago). They were traditionally classed as amphibians but are more properly called anamniote tetrapods. Temnospondyls were mostly from 0.5 to 3 m long, but some giants reached 6 m, and they occupied various terrestrial, amphibious, and aquatic niches [1–5]. Temnospondyls flourished in the Carboniferous and Permian and declined in diversity in the Late Permian. The great mystery is that, although like so many other groups, they were hit hard by the Permian–Triassic mass extinction (PTME; 252 Ma), they diversified rapidly in the post-extinction ‘hothouse’ of the Early Triassic [6–8], giving rise to numerous lineages (figure 1a) that continued at diminishing diversity through the remainder of the Triassic, with a few species surviving into the Jurassic and Cretaceous. Early Triassic temnospondyls have been found in India, Pakistan, Greenland, Norway, Australia, South Africa and the USA [2,11–13], representing a mix of freshwater, terrestrial and, rarely, marine conditions: this highlights their Early Triassic diversity peak, which exceeded that of their previous diversity peak in the Early Permian [7].

The Early Triassic was a time of repeated volcanism leading to phases of global warming, aridification, reductions in atmospheric oxygen, acid rain and widespread wildfires [14], even creating conditions so hostile that the tropics became devoid of animal life; this ‘tropical dead zone’ (TDZ) [15] drastically impacted the distributions of both marine and terrestrial organisms [16–18]. Only when global climates stabilized, in the Middle Triassic, did other forms of life recover [19], but temnospondyls began their long decline. Their brief post-PTME flourish has seen temnospondyls labelled as ‘disaster taxa’ [6,7,13,20]. Nonetheless, temnospondyls remained prominent members of later Triassic faunas, even as climates fluctuated between humid equatorial monsoonal conditions and hot, dry, seasonal conditions [21].

How and why did temnospondyls undergo this second, Mesozoic, diversification? We explore three themes to seek answers. Were these earliest Triassic temnospondyls unusually small, an adaptation to post-extinction grim environmental conditions termed the ‘Lilliput effect’?—previous studies are equivocal [3,8,13]. Second, as ‘disaster taxa’ (early diversifying survivors of a mass extinction), we might expect that they would show some different adaptations from relatives that lived in more normal times, or that they would differ substantially in size, or occupy specific habitats and/or niches. Third, we might expect some evidence that these disaster taxa were successful in a particular region of the world where physical conditions and coeval faunal elements were favourable. Overlying all such studies are concerns about incompleteness of the data, and we consider this issue too.

The peak in temnospondyl diversity was identified in previous work [7,8] as well as expansion of morphospace represented by differing skull shapes [22–25]. Here, we explore the body size, feeding ecology, disparity, and biogeographic distributions of temnospondyl morphology through the Triassic. We apply a variety of computational approaches in morphometrics, and macroevolutionary and palaeobiogeographic modelling to establish an appropriate numerical regime in which to test hypotheses for temnospondyl success and decline following the PTME. Our aim is to understand changes in temnospondyl ecospace occupation through this time, the phylogenetic structure of such changing patterns, and overall global palaeogeographic distribution as the clade expanded and shrank in the early Mesozoic.

## 2. Material and methods

### 2.1. Sampling

We compiled a list of all temnospondyl taxa from the late Permian–Early Jurassic (259.1−174.1 Ma), using an updated dataset based on [26]. We found that 99 species from our initial list of 120 possessed sufficient cranial materials for analysis, based on specimen photographs, reconstructions and descriptions from the literature. Temporal ranges for each taxon were extracted and updated according to the latest literature. Geographical occurrence data were also extracted from the literature and palaeolatitudinal data downloaded from the Paleobiology Database on 2nd August 2022 (PBDB; http://www.paleodb.org/).

We focus on the Stereospondyli, as their evolution encompasses the latest Permian and early Mesozoic (figure 1a). We therefore exclude dissorophoids and lissamphibians, each with two species in the Early Triassic. Stereospondyl diversity is dominated by two classic subclades, Capitosauria and Trematosauria [27], although there has been debate about whether rhytidosteids and brachyopoids are trematosaurs [4]. Therefore, we further examine evolutionary patterns across capitosaurs and trematosaurs, as well as smaller, well-established subclades within these groups to better clarify the potential ecomorphological diversity of temnospondyls through the Permian–Triassic transition and early Mesozoic. This study includes 36 capitosaur species and 39 trematosaur species. We further subdivide the Trematosauria into smaller subclades: Brachyopoidea (9), Rhytidosteidae (5), Plagiosauridae (6) and Metoposauroidea (6). Four late Permian and one early Triassic temnospondyl were grouped as ‘basal temnospondyls’ (*Nigerpeton ricqlesi, Dvinosaurus primus, Peltobatrachus pustulatus, Saharastega moradiensis* and *Thabanchuia oomie*).

### 2.2. Phylogenetic topology

We constructed an informal supertree of all 389 temnospondyl species, as there is no complete published tree, whereas there are multiple recent publications that cover large portions of the overall clade. The tree extends back to the origin of temnospondyls in the Carboniferous to ensure the correct tree geometry for those that span our sampling interval (Late Permian–Early Jurassic). We used a widely cited tree scaffold [28], based on an expanded character matrix of an earlier work [29] and added taxa using Mesquite (v.3.5.1; [30]). When selecting phylogenies for the supertree construction, preference was given to recent analyses featuring taxon- and character-rich data matrices. Major subclades and grades which we used to expand the scaffold are Dvinosauria [31], early rhachitome taxa [32], Dissorophoidea [33–37], early stereospondylomorphs and stereospondyls [4], Capitosauria [38,39] and Trematosauria [40–45]. Taxa that have never been included in a phylogenetic analysis were added based on alpha taxonomic opinion, such as that a new species belonged to a genus or family-level clade already in the tree. Adding taxa based on taxonomies for subsequent phylogenetic comparative analyses is warranted if care is taken when placing the taxa [46].

### 2.3. Phylogenetic time-scaling

Taxa were dated to stratigraphic substage level through the time range from Wuchiapingian to Pliensbachian (259.5−184.2 Ma). Age ranges were based on the most recent information available for each taxon, with a focus on dating the geological formations in which each taxon occurred. Absolute ages for geological stages were based on the 2023/2024 version of the International Chronostratigraphic Chart [47]. Polytomies were randomly resolved prior to time-scaling, generating 100 input trees.

We time-scaled our phylogeny using the cal3 approach, with the bin_cal3TimePaleoPhy function of the paleotree package (v.3.4.5) [48,49] in the R environment (v4.3.2) [50]. The cal3 method is a probabilistic ‘*a posteriori*’ time-scaling approach that draws divergence times under a birth–death sampling model based on *a priori* known rates of branching, extinction and sampling [49,51,52]. Since calculating sampling rates is not trivial [52,53], we obtained the instantaneous sampling rate from a uniform distribution bounded by the lowest (0.018 lineages per million years (lmy^−1^)) and highest estimates (0.18 lmy^−1^) reported in the literature for Devonian tetrapods and Mesozoic dinosaurs [52,54]. These sampling rate estimates were then used to calculate the extinction and origination rates, following [55]. The time of observation was treated as uncertain and was randomly sampled between the first and last appearance times (dateTreatment: randObs), the step size of increments used in the function to set node ages was set to 0.001 (step.size = 0.001), and the probability of inferring ancestor-descendant relationships was set to 0 (anc.wt = 0). The cal3 algorithm tends to produce several zero-length branches [56] which can be problematic for some phylogenetic comparative methods. To overcome the problem of zero-length branches, we added 0.0001 Myr (= 100 yr) to all branches with a length of zero [52,57]. Subsequent analyses were run on a randomly chosen tree from the time-scaled complete tree, pruned to the taxa for which morphometric data were available.

### 2.4. Temnospondyl size through time

We used basal skull length as a proxy for body size [58] to provide a synoptic overview of temnospondyl size through time as this measurement is widely available across fossil taxa, offering a broadly applicable measure of relative size. We grouped taxa on their midpoint appearance ((FAD+LAD)/2) and then calculated summary statistics for skull length measurements across substages for our entire dataset and for three subclades (Trematosauria, Capitosauria and ‘Basal Stereospondyls’) that have enough data and whose lineages cross the Permian–Triassic boundary (PTB). Mean log skull length (mm), s.d. and 95% confidence intervals were calculated—using the s.e. formula, for each time interval. The plots were made using the R deeptime [59] and ggplot2 [60] packages.

### 2.5. Morphometric analyses

We used a mixture of discrete and continuous functional characters encompassing measurements of the teeth and jaws that are informative about feeding habits and broader ecology (see [61]; electronic supplementary methods). Morphometric analysis using these characters enables quantitative discrimination of subtle differences in functional morphology across a wide array of taxa, revealing potential patterns of ecological variation. Measurements were taken in the Fiji bio-imaging program [62] to incorporate the relative scales of each image and allow accurate absolute measurements. Our initial list of 33 functional characters was reduced to 19 characters whose functional significance had been determined in previous literature [61]. These characters denoted distinct overall skull shapes and specific feeding modes (figure 1b) that could be measured confidently for most included taxa. Basal skull length was used as a representative of body size [58].

We created a Euclidean distance matrix from the functional characters in the R package FactoExtra (v.1.0.7) [63] and subjected this matrix to a principal coordinate analysis (PCO) using the R stats package (v.3.6.2) [50] to map the primary axes of morphological diversity (disparity). Despite pruning characters that could not be widely coded for many taxa, our remaining functional characters still included some missing data for some taxa. For missing trait data, the PCO used the mean value for that trait, a conservative approach that exaggerates overall similarities between taxa.

We visualize disparity through the first two principal components (PCs), which represent the majority of variation, to create morphospaces. The morphospaces were subdivided by stage level to show changes in morphospace occupation through time. All plots were created in R using the ggplot2 package [60].

### 2.6. Temporal patterns of disparity

Disparity through time was explored using the dispRity (v.1.8) [64] package to calculate the sum of variance (SOV) for taxa present in each substage using phylogenetic time-slicing to incorporate unsampled lineages [65]. We further dissected trends in disparity by distinguishing between subclades, continental location (Gondwana and Laurasia) and palaeolatitudes. SOV was calculated using 2000 bootstraps and rarefaction to account for sample size biases. SOV was chosen over sum of ranges (SOR) as we were interested in the spread of taxa within the morphospace to ascertain functional morphology of the temnospondyl skulls. SOV is a variance-based approach while SORs quantify morphovolume that can be affected by biases in clade-level time bins, where for example there are too few taxa [66,67]. We use morphovolume to include variation along all axes in multidimensional hyperspace, rather than simply the area on two axes or volume on three axes. These trends in SOV were plotted using the R geoscale [68] and strap [69] packages.

We tested for significant shifts in morphospace occupation using Wang’s Permutational analysis [70] and PERMANOVA, which was applied in PAST (v.4.10) [71]. We compared the observed and expected differences between Laurasia and Gondwana, and differences between time bins. Temporal comparisons through the Rhaetian, Hettangian, Sinemurian and Pliensbachian could not be assessed because sample sizes in these time bins were too small.

### 2.7. Macroevolutionary modelling

We compared patterns of SOV disparity through time with estimated models of trait evolution using the DispRity (v.1.8) [64] package. We tested whether patterns of disparity in all temnospondyls, capitosaurs, metoposauroids, trematosaurs and basal stereospondyls followed either Brownian motion, Ornstein–Uhlenbeck, Trend, Stasis or Early Burst models of evolution [72–74]. Identifying which evolutionary model applies to all the data, and to subclades, can help interpret the patterns of temnospondyl diversity and disparity in the Early Triassic. In adaptive radiations, it has often been expected that taxa would show an initial acceleration, and then gradual slowdown in diversification—an ‘early burst’ [75]. If a specific ecomorphology were highly successful, then we might expect either greater selection for that morphology driving a trend model of directed evolution, or once achieved, stabilizing (Ornstein–Uhlenbeck) selection to maintain that morphology [73].

### 2.8. Phylogenetic biogeography estimation

We used a phylogenetic estimation approach to investigate basic biogeographic patterns of temnospondyl diversification across Laurasia, Gondwana and equatorial regions through the late Permian to Early Jurassic. However, as the rates of change for morphological trait and biogeographic range shifts differ, typical models of discrete trait estimation [76] do not realistically model biogeographic range evolution [77]. Therefore, we used the specialized biogeographic estimation R package, BioGeoBEARS [78] to analyse temnospondyl biogeographic patterns using a maximum likelihood approach. This approach models geographic range evolution stochastically along the branches of a phylogeny, reflecting how dispersal and local extinctions may shape range evolution between ancestors and their descendants [79] and how time can affect the occurrence of biogeographic events [78].

We applied the default dispersal-extinction cladogenesis model (DEC) [77] and a likelihood version of dispersal-vicariance analysis (DIVALIKE) [80]. The DEC model allows for sympatry but little vicariance, whereas the DIVALIKE model permits widespread vicariance, but not subset sympatry [81]. While BioGeoBEARS includes options to incorporate ‘founder event’ rapid dispersals into its modelling, it has been noted that this model tends to overemphasize rapid range changes [82] and so, following the precedent of recent BioGeoBEARS analyses [83], we did not apply it here. We set the maximum range size parameter to three, which denotes a global distribution across all geographic zones (Laurasia, Equatorial and Gondwana). We also followed the protocol of Poropat *et al*. [84] when dealing with uncertain geographic barriers, by using a dispersal constraint value of 0.5, meaning dispersal is neither particularly easy nor difficult. Further, we opted not to conduct a time-stratified analysis for three reasons: (i) our focus is largely on the short timespan across the PTB; (ii) our data through this time are not resolved to very small time bins; and (iii) there actually is little evidence for major palaeogeographic change at global scale through this time. The model of best fit was identified using the natural log of the process likelihood (LnL) and the Akaike information criterion (AIC).

## 3. Results

### 3.1. Body size through time

**Figure 2.**
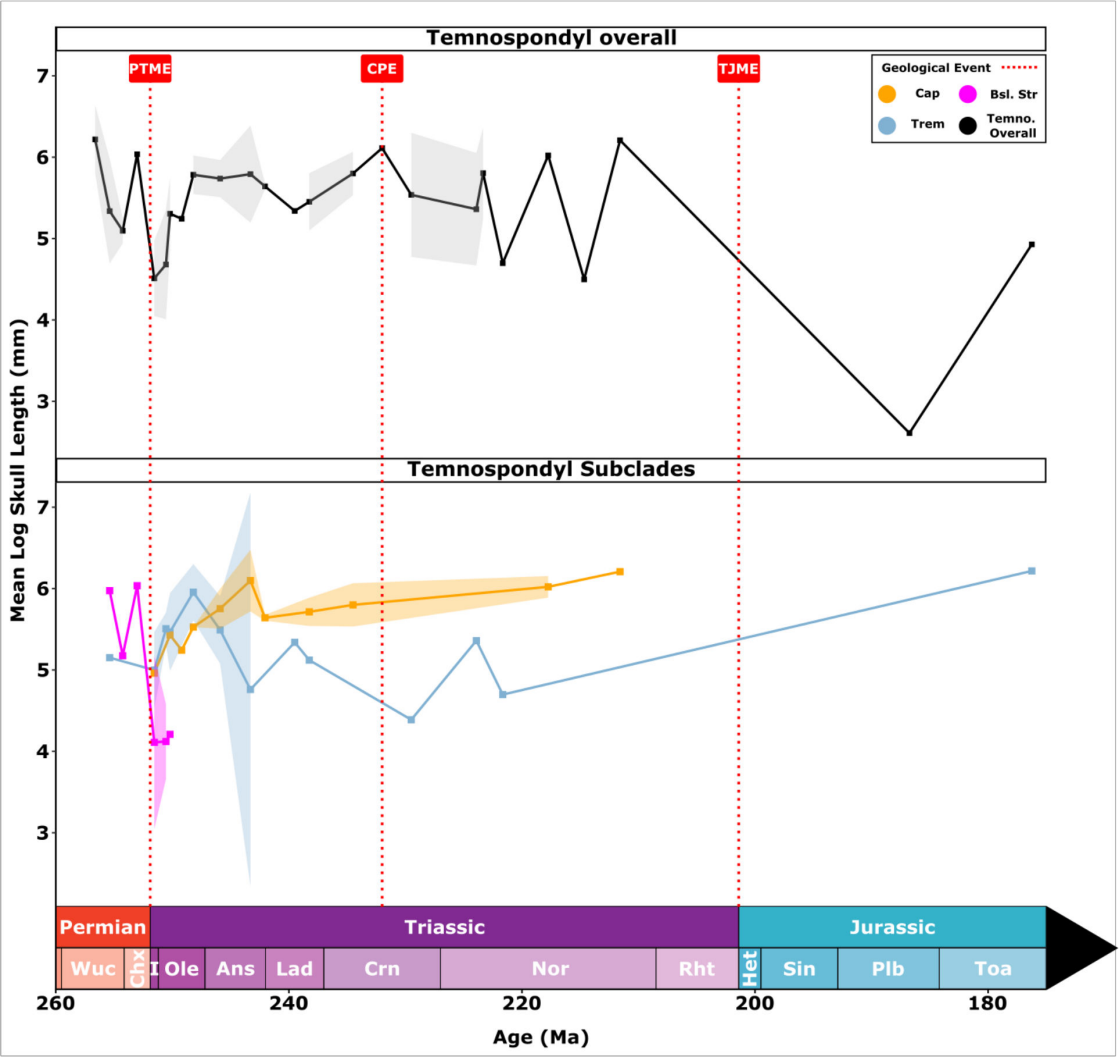
Mean Log skull length (mm) as a proxy for body size for temnospondyls from the Late Permian to the Early Jurassic, 160−175 Ma. For temnospondyls overall (*a*), there is a small dip in mean size, they reach near pre-extinction distributions quite rapidly by the Olenekian, following a rapid dip across the PTB. (*b*) Temnospondyl subclades during the same interval. Basal stereospondyls exhibit a comparatively sharp decrease in size around the PTB before disappearing. Trematosauria and Capitosauria conversely show a gradual increase in size reaching pre-extinction levels by the Olenekian and Anisian respectively. Areas around lines represent confidence intervals, which cannot be calculated after the Mid Norian due to low sample sizes. Differences in Jurassic body sizes reflect inclusion of lissamphibian *Notobatrachus* in upper diagram, exclusion in lower diagram. Clade abbreviations: Cap, Capitosauria; Trem, Trematosauria; Bsl. Stereo, Basal stereospondyls; Temno. Overall, Temnospondyls overall. Event abbreviations: CPE, Carnian Pluvial Episode; PTME, Permian–Triassic mass extinction; TJME, Triassic–Jurassic mass extinction. Stage abbreviations: Ans, Anisian; Cap, Capitanian; Chx, Changhsingian; Crn, Carnian; Het, Hettangian; I, Induan; Lad, Ladinian; Nor, Norian; Ole, Olenekian; Plb, Pliensbachian; Rht, Rhaetian; Sin, Sinemurian; Toa, Toarcian; Wuc, Wuchiapingian.

Overall temnospondyl body size trends were generally quite consistent through the Early–Middle Triassic, with a long-term, moderate decrease in average size from the Wuchiapingian to Toarcian (figure 2a). Across the PTB, temnospondyls experienced a sharp drop in average sizes, but returned to roughly pre-extinction mean sizes from the Olenekian to middle Carnian. Insufficient data from the middle Norian onwards means that patterns for the latest Triassic and Early Jurassic should be viewed with caution. Body size changes of temnospondyl subclades show more complex patterns (figure 2b). ‘Basal stereospondyls’ saw an acute decline in their mean body size across the PTB. Trematosaur mean sizes also declined across the boundary, but much less sharply. Both trematosaurs and capitosaurs saw strong increases in mean size through the Early Triassic, reaching maximum sizes at different times, trematosaurs in the Olenekian, capitosaurs in the Anisian. Their size trajectories diverged, however, with trematosaurs showing variable sizes through the rest of the Triassic, and capitosaurs increased in size by a small amount. At the onset of the Norian, trematosaurs show an increase in body size, which likely represents the emergence of the Metoposauroidea. Body size in Tematosauria trends upwards into the Early Jurassic with the emergence of chigutisaurids like *Siderops*.

### 3.2. Temnospondyl morphological diversity

**Figure 3.**
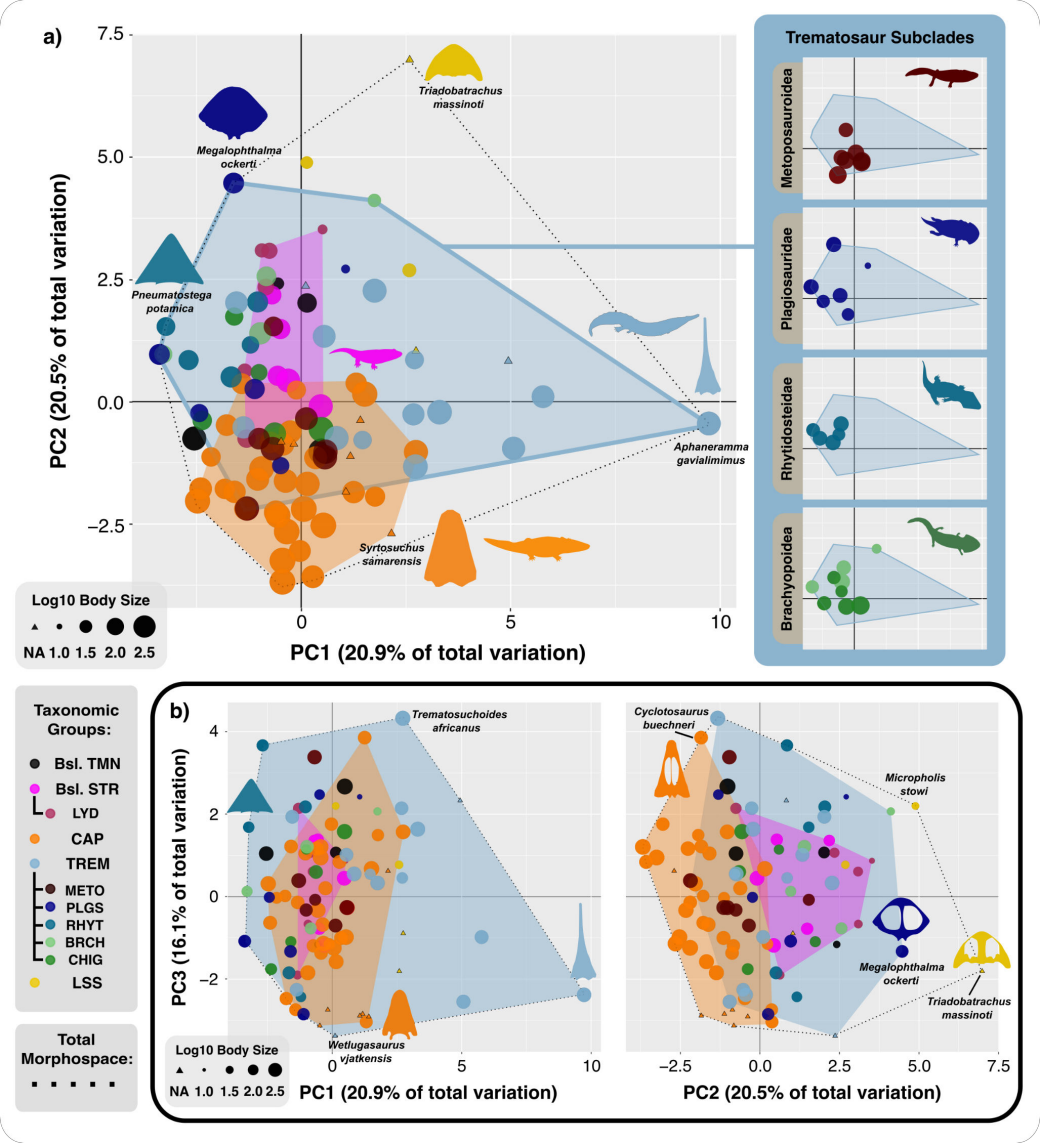
Morphospaces of Late Permian to Early Jurassic temnospondyls, showing PC1 versus PC2 (*a*), PC1 versus PC 3 (*b*) and PC2 versus PC3 (*c*). PC1 (20.9% of total variation) represents largely snout length, with shorter and more broad-snouted forms on the left, and long-snouted skulls on the right. PC2 (20.5% of variation) summarizes the length–width ratio of the interpterygoid vacuities and skull width across the orbits. The skull of *Triadobatrachus*, with elliptical, anteriorly positioned orbits, is at the top of the morphospace. There are no skulls in the top, right-hand corner, representing hypothetical long-snouted temnospondyls with broad posterior skulls. PC3 (16.1% of variation) shows the shapes of the premaxilla, orbits and the orbit position relative to the nares and parietal foramen. There is an equal spread of taxa along PC3. Trematosauria has been subdivided to show its constituent clades, Plagiosauridae and Chigutisauridae appear to be the more varied across PC1 and PC2, while Rhytidosteidae and Metoposauroidea are more conservative. Clade abbreviations: Brch, Brachyopidae; Bsl. Str, Basal stereospondyls; Bsl. Tmn, Basal temnospondyls; Cap, Capitosauria; Chig, Chigutisauridae; Lss, Lissamphibia; Lyd, Lydekkerinidae; Meto, Metoposauroidea; Plgs, Plagiosauridae; Rhyt, Rhytidosteidae; Trem, Trematosauria. Silhouette images from Phylopic (https://www.phylopic.org: Dmitry Bogdanov (*Metoposaurus*, *Lydekkerina*, *Batrachosuchus*, *Pelorocephalus*, *Trematosaurus*) all CC0 3.0; Steven Traver (*Dendrobates*) CC0 1.0). Nix Draws Stuff/Nix illustration (*Eryops*) CC0 4.0. Silhouette images from Wikimedia Commons (https://commons.wikimedia.org: Smokeybjb (*Eolydekkerina magna*, *Deltasaurus kimberleyensis*) all CC0 3.0; Nobu Tamura (*Gerrothorax* BW and *Wetlugasaurus* BW) all CC0 3.0). Skull vector graphics vectorized by AM: (*Cyclotosaurus*, *Wetlugasaurus*, *Pneumatostega* [1]; *Aphaneramma* [85]; *Triadobatrachus* [9]; *Megalophthalma* [43]).

The morphospace (figure 3a) discriminates the classic short-snouted and long-snouted temnospondyl morphotypes along PC1 (20.9% of total variation), with relatively short and broad-snouted forms located in the more negative areas of PC1, and longer snouted skulls in the positive region, *Aphaneramma* being the most extreme example. PC2 (20.5% of variation) summarizes the length–width ratio of the interpterygoid vacuities and skull width across the orbits. The skull of *Triadobatrachus*, with elliptical, anteriorly positioned orbits, is located at the top and centre of the morphospace. There are no skulls in the top, right-hand corner, representing hypothetical long-snouted temnospondyls with broad posterior skull dimensions. Note that sizes of narrow skulls, in the lower half of the morphospace, are varied, whereas the more extreme, broad skulls (top left) tend to be from smaller animals. PC3 (16.1% of variation) summarizes the shapes of the premaxilla, orbits and the orbit position relative to the nares and parietal foramen (figure 3b). Here, there is an equal spread of taxa along the length of PC3, from negative to positive values. Further, the top right-hand spaces are empty, corresponding to hypothetical skulls with broad posterior portions (PC2) and long snouts (PC1).

On PC1 and PC2 (figure 3b), the greatest density of taxa occurs in the lower left of the morphospace around the most species-rich clade in this analysis, Capitosauria. This area is also occupied by other taxonomic groups, including basal temnospondyls, Metoposauroidea, Trematosauria, and Brachyopoidea. The skulls of all these temnospondyls are large and anteriorly roughly parabolic, with a rounded premaxilla. Late Permian *Nigerpeton* shares the same morphospace area as Early Triassic *Wetlugasaurus* and Late Triassic *Metoposaurus*, suggesting convergence among these three.

Trematosaurs show the greatest variance along PC1 and 2, ranging from short- to long-snouted forms across the entire width represented by PC1, and most of the span of PC2 as well (figure 3a). In the Early Triassic, capitosaurs and trematosaurs spanned the entire range from short- to long-snouted forms, the latter being adapted to fast, but weaker bites used to catch small, fast-moving prey [86,87]. Trematosauria and Capitosauria possess the greatest variance along PC3, showing a range of premaxilla and orbital variations. However, the Metoposauroidea as a clade aggregate closely in the centre of the morphospace, with the exception of *Arganasaurus* (figure 3b). Higher values along PC2 correspond to many of the Amphibamiformes and some basal stereospondyls, and generally these species are smaller than the trematosaurs and capitosaurs, which have more ellipsoid interpterygoid vacuities. These smaller taxa have more oval-shaped interpterygoids (figure 3b).

### 3.3. Disparity through time

**Figure 4.**
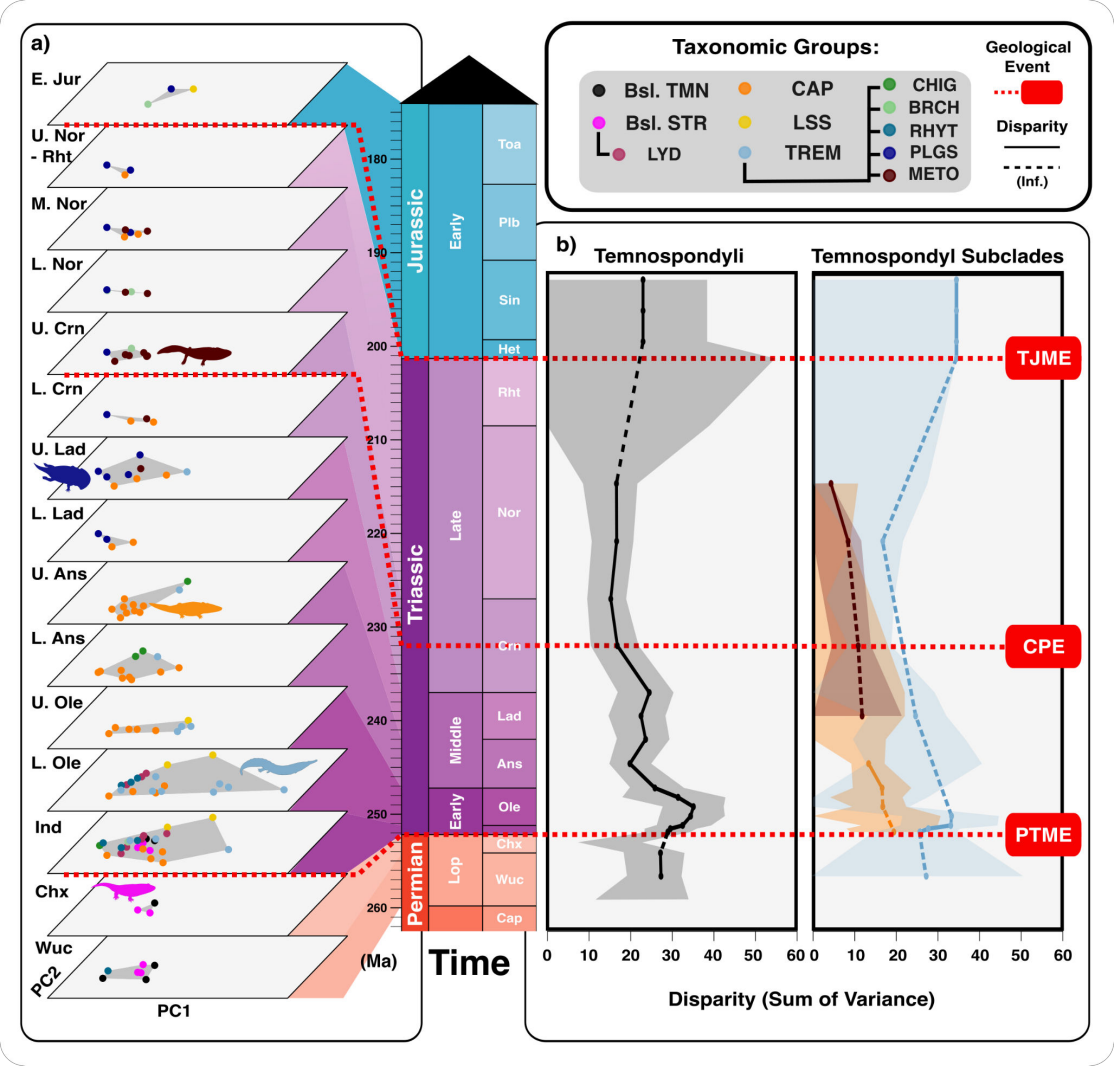
Evolution of temnospondyl morphospace occupation through time, from Late Permian to Early Jurassic. (a) varying morphospace occupation through time, from Late Permian to Early Jurassic, highlighting the largest occupation of morphospace in the Induan and lower Olenekian, with further, smaller expansions in the Anisian, upper Ladinian, and upper Carnian; one morphospace area to the left seems to remain constant through time, with expansions time and again along PC1. (b) calculated and inferred (inf.) disparity through time for all Temnospondyli and for Capitosauria, Trematosauria, and Metoposauroidea, showing generally reducing levels of disparity through the Triassic from the Early Triassic high point; confidence intervals increase through time as sample sizes reduce. Clade abbreviations: Brch, Brachyopidae; Bsl. Str, Basal stereospondyls; Bsl. Tmn, Basal temnospondyls; Cap, Capitosauria; Chig, Chigutisauridae; Lss, Lissamphibia; Lyd, Lydekkerinidae; Meto, Metoposauroidea; Plgs, Plagiosauridae; Rhyt, Rhytidosteidae; Trem, Trematosauria. Event abbreviations: CPE, Carnian Pluvial Episode; PTME, Permian–Triassic mass extinction; TJME, Triassic–Jurassic mass extinction. Stage abbreviations: Ans, Anisian; Cap, Capitanian; Chx, Changhsingian; Crn, Carnian; H, Hettangian; Ind, Induan; Lad, Ladinian; Nor, Norian; Ole, Olenekian; Plb, Pliensbachian; Rht, Rhaetian; Sin, Sinemurian; Toa, Toarcian; Wuc, Wuchiapingian. Silhouette images from Phylopic (https://www.phylopic.org: Dmitry Bogdanov (*Metoposaurus*, *Lydekkerina*, *Trematosaurus*) all CC0 3.0). Silhouette images from Wikimedia Commons (https://commons.wikimedia.org: Nobu Tamura (*Gerrothorax* BW and *Wetlugasaurus* BW) all CC0 3.0).

When the data are divided into time bins, disparity can be seen to change substantially through the Late Permian to Early Jurassic interval (figure 4a). Diversity and disparity vary substantially through the Triassic, to some extent matching mass extinctions and Triassic climatic events.

Late Permian temnospondyls occupy a small area of morphospace at the lower left, 10% of the area occupied in the Induan, and their morphospace spiked immediately following the PTME, with the radiation of trematosaurs, basal stereospondyls and capitosaurs (figure 4a). Some early diverging trematosaurs capitalized on the longirostrine ecomorphology, while many others clustered around large parabolic skull shapes. The long-snouted trematosaurs went extinct in the Anisian, leading to a considerable and lasting reduction in overall temnospondyl morphospace occupation (figure 4a). The Middle Triassic saw a brief diversification of large mastodonsaurid capitosaurs but they disappeared towards the end of the Ladinian with more derived metoposaurids occupying this vacated morphospace (figure 4a). Through the Middle Triassic disparity fluctuates, stabilizing during the CPE, an event that facilitated a minor resurgence in temnospondyl diversity, though overall diversity was at its lowest.

In measuring disparity through time with SOV, robust results could be obtained only for all temnospondyls, but more diffuse results for capitosaurs, trematosaurs and metoposauroids because of smaller sample sizes (figure 4b). There is a steady decline in temnospondyl disparity throughout the Triassic, and following the Olenekian the decline curve is sharp and continues (at a slower rate) until the Norian (figure 4b). Metoposauroidea (red) shows a less pronounced pattern with steady disparity from late Ladinian to middle Norian. Other clades did not show coherent patterns, but the predicted line for Trematosauria suggests a similar trend in the middle Triassic, which is one of decline (figure 4b, blue). Disparity remained low through the Late Triassic and onwards into the Jurassic, and these time bins are sparsely populated, so trends following the middle Norian cannot be reliably interpreted (figure 4b). Through time, there was an overall reduction in variation of PC1 and PC2 (figure 4a), but tests for significant changes in disparity between time bins through PERMANOVA yielded no significant results (electronic supplementary material, table S2).

**Figure 5.**
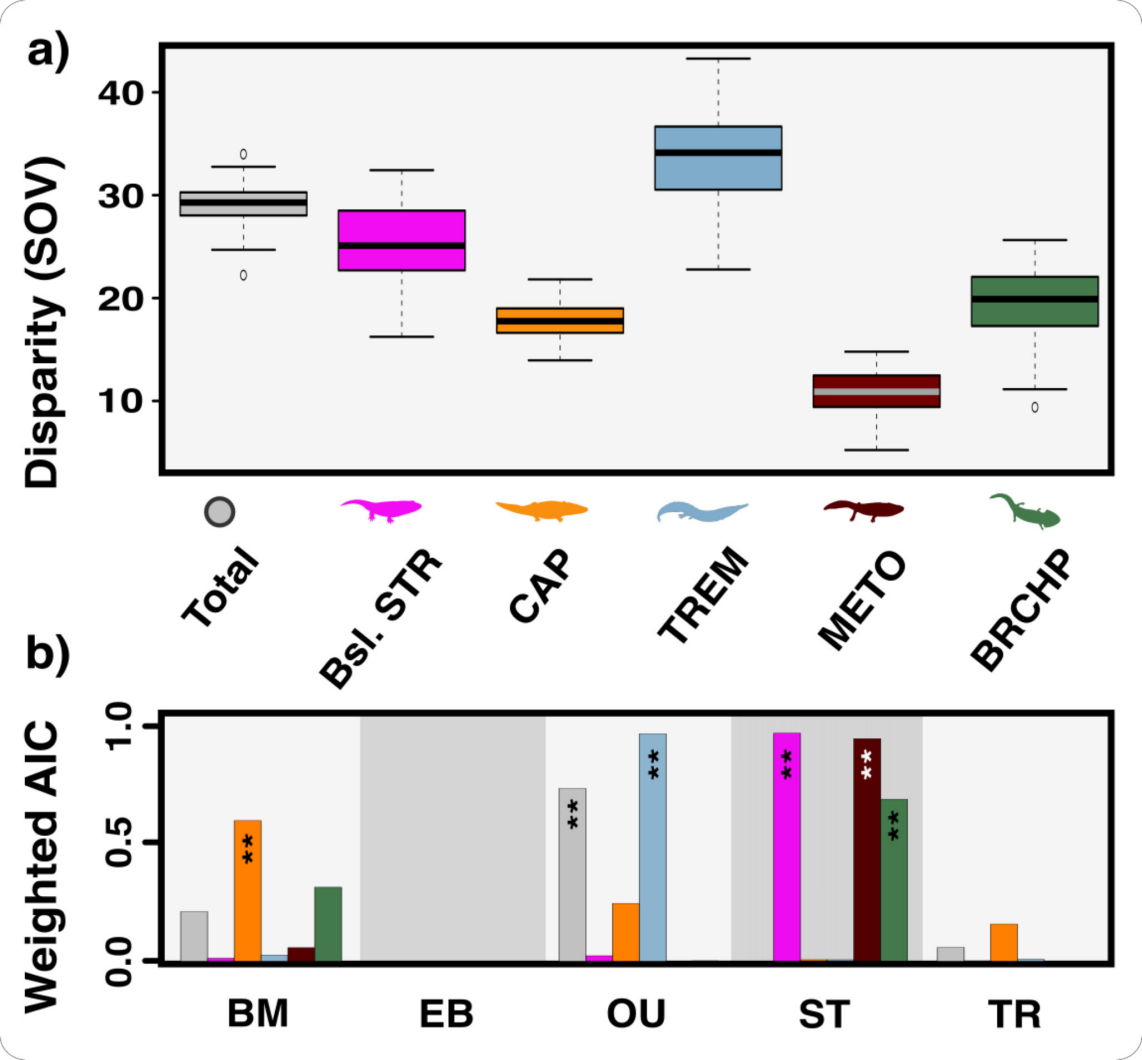
Temnospondyl disparity and evolutionary modelling. (a) total disparity (SOV, sum of variances) for the key groups showing conservative disparity ranges especially in Capitosauria, Metoposauroidea and Temnospondyli overall, while Trematosauria differ significantly from all other clades. (b) support for different evolutionary models for temnospondyl disparity diversification (BM, Brownian motion; EB, early burst; OU, Ornstein–Uhlenbeck; ST, stasis; TR, trend), according to weighted Akaike information criterion (AIC). Asterisks represent models with strongest support for each clade. Clade abbreviations: Brchp, Brachyopoidea; Bsl. Str, Basal stereospondyls; Bsl. Tmn, Basal temnospondyls; Cap, Capitosauria; Meto, Metoposauroidea; Trem, Trematosauria. Silhouette images from Phylopic (https://www.phylopic.org: Dmitry Bogdanov (*Metoposaurus*, *Lydekkerina*, *Batrachosuchus*, *Pelorocephalus*, *Trematosaurus)* all CC0 3.0). Silhouette images from Wikimedia Commons (https://commons.wikimedia.org: Nobu Tamura (*Wetlugasaurus* BW) all CC0 3.0).

Temnospondyls overall show relative conservatism in skull shapes through the sampled time interval, a feature that is shared with Capitosauria, Brachyopoidea and especially Metoposauroidea. It is also important to note that these three subclades share similar morphospace areas, though at different times, the Capitosauria in the Induan–Anisian, the Brachyopoidea in the Ladinian, and the Metoposauroidea in the late Carnian (figure 4b). Trematosauria show the greatest range in disparity compared to all other groups, which is likely due to the differences in skull morphology and by extension feeding habits within this group. Basal stereospondyls show fairly large disparity, occupying a broad central area of morphospace, adjacent to the core area of morphospace occupied by most later Triassic temnospondyls (figures 1 and 5a). Wang’s Permutation tests (electronic supplementary material, table S1) identify several significantly different pairwise comparisons: Capitosauria and Metoposauroidea are significantly different from every other group except each other, though Metoposauroidea is significantly different from Brachyopoidea (*p* = 0.011), whereas Capitosauria is not (*p* = 0.365).

Evolutionary modelling shows strongest support for an Ornstein–Uhlenbeck model of morphological trait evolution in temnospondyls overall, indicating that phenotypic evolution is constrained to revert to a central tendency. A similar result was obtained for Trematosauria, highlighting a propensity towards stabilizing selection in their evolution. We found that the stasis model better captured metoposaurid, basal stereospondyl and brachyopoid evolution (figure 5b). There was little to no support for either the Trend or Early burst models.

### 3.4. Palaeogeography

**Figure 6.**
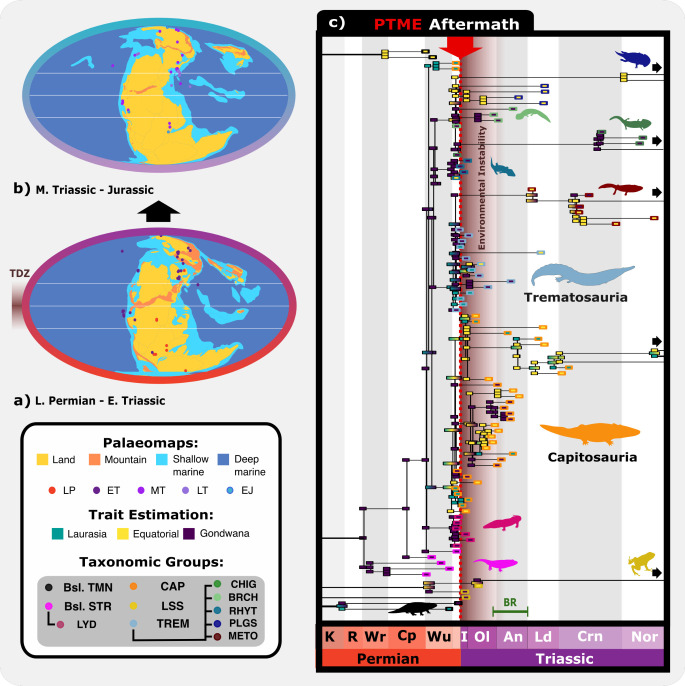
Patterns of geographic evolution of the temnospondyl clades. (a, b) geographic occurrences of temnospondyls, colour coded by time interval (LP, Late Permian; ET, Early Triassic; MT, Middle Triassic, LT, Late Triassic, EJ, Early Jurassic). Shading highlights the possible ‘tropical dead zone’ (TDZ). (c) ancestral state reconstruction for temnospondyl biogeography through the Permian–Triassic transition. As many basal nodes unresolved as we used only three geographic zones (equatorial and temperate north and south—Laurasia and Gondwana). After the PTME, the Lydekkerinidae diversified in Gondwana, the Capitosauria likely originated in Laurasia and diversified in the Middle Triassic and then moved into Gondwana (especially *Paracyclotosaurus*), the Cyclotosauria dominated equatorial regions in the Carnian and Norian, the Trematosauria probably originated in Gondwana, as did the Plagiosauridae, Rhytidosteidae, Chigutisauridae and Brachyopidae, the Metoposauroidea were an offshoot of the Laurasian trematosaurs. Event abbreviations: PTME, Permian–Triassic mass extinction. Clade abbreviations: Brch, Brachyopidae; Bsl. Str, Basal stereospondyls; Bsl. Tmn, Basal temnospondyls; Cap, Capitosauria; Chig, Chigutisauridae; Lss, Lissamphibia; Lyd, Lydekkerinidae; Meto, Metoposauroidea; Plgs, Plagiosauridae; Rhyt, Rhytidosteidae; Trem, Trematosauria. Timebin abbreviations: An, Anisian; Cp, Capitanian; Crn, Carnian; I, Induan; K, Kungurian; Ld, Ladinian; Ol, Olenekian; Nor, Norian; R, Roadian; Wr, Wordian; Wu, Wuchiapingian. Palaeomaps from GPlates (https://www.gplates.org). Silhouette images from Phylopic (https://www.phylopic.org: Dmitry Bogdanov (*Metoposaurus*, *Lydekkerina*, *Batrachosuchus*, *Pelorocephalus*, *Trematosaurus*) all CC0 3.0; Steven Traver (*Dendrobates*) CC0 1.0). Nix Draws Stuff/Nix illustration (Eryops) CC0 4.0. Silhouette images from Wikimedia Commons (https://commons.wikimedia.org: Smokeybjb (*Eolydekkerina magna*, *Deltasaurus kimberleyensis*) all CC0 3.0; Nobu Tamura (*Gerrothorax* BW and *Wetlugasaurus* BW) all CC0 3.0).

Temnospondyls enjoyed a global distribution through the late Permian–Early Jurassic, although their prevalence declined drastically through time (figure 6a,b). Late Permian taxa appear to be more widespread in the Southern Hemisphere, whereas most Triassic diversity is found in the Northern Hemisphere. Consequently, it is unsurprising that phylogenetic estimation of ancestral biogeography suggests that most Induan temnospondyls diversified prior to the PTME in the Southern Hemisphere (figure 6c) [61], electronic supplementary material, figures S2). Basal stereospondyls such as *Peltobatrachus* and *Uranocentrodon* became extinct prior to the PTME. However, the Lydekkerinidae radiated in Gondwana, enjoying a brief burst of diversification before going extinct around 251 Ma in the Early Triassic.

A surprising discovery, because of the existence of the TDZ, was that the Early Triassic diversity spike occurred in equatorial regions, both for all Temnospondyli and for Capitosauria (figure 6c), with the pattern of node and tip states suggesting movement back and forth from the tropics. Temnospondyl diversification in the Middle Triassic is also largely based in the tropics, with a general shift towards the Southern Hemisphere for most remaining temnospondyls that continued into the Late Triassic [61], (electronic supplementary material, figures S2). Our PERMANOVA showed a significant difference (*F* = 2.6, *p* = 0.0001) between the temnospondyl faunas of Gondwana and Laurasia.

Some smaller subclades had apparently more exclusive geographic distributions, such as Chigutisauridae (Gondwana), Wetlugasauridae (Laurasia) and Lydekkerinidae (Gondwana), but otherwise, the clades Trematosauria, Capitosauria, Metoposauroidea and Rhytidosteoidea are distributed across all three geographic zones (figure 6c). Amphibamiformes appeared in the Induan with *Triadobatrachus and Czatkobatrachus*, but the sparse fossil record means there is a considerable gap between these early diverging species and the Toarcian *Notobatrachus.* Capitosaurs underwent broad radiations encompassing all regions in the Changhsingian, Induan and Olenekian, highlighting their success through the Permian–Triassic transition (figure 6c) [61] (electronic supplementary material, figure S2). They showed some modest diversification in the Middle and Late Triassic but by this time, capitosaur diversity had been reduced to just one equatorial clade, the cyclotosaurids.

Greater and more sustained diversity levels are seen in the Trematosauria. They show an immense diversification across the PTB and appear to have two distinct lineages: one diversifying in Laurasia and the other in Gondwana. The first branch leads to the distinctive longirostrine condition seen in *Aphaneramma* and *Wantzosaurus,* and this skull shape flourished for a similarly short time in the ‘basal stereospondyls’, first in Laurasia and then in Gondwana. This lineage enjoyed a brief resurgence in the Late Triassic with the radiation of the Metoposauroidea. The second lineage was Gondwanan, diverging from other Trematosauria before the PTB and encompasses subclades that prevailed through a longer span of the Triassic; rhytidosteids, brachyopids, plagiosaurids and chigutisaurids all show considerable branching at their stems in the Changhsingian (figure 6c) [61] (electronic supplementary material, figures S2). However, each clade appears to have thrived at different times and some in different regions through the Triassic. The rhytidosteids and brachyopids were abundant in the Early Triassic across southern and equatorial regions, whereas plagiosaurids were more commonplace in the Middle Triassic and only in equatorial regions. The chigutisaurids thrived in the Late Triassic and were the only temnospondyls to survive beyond the Triassic but were limited to Gondwana. The chigutisaurids and metoposauroids were the only trematosaurs to enjoy radiations following the Early Triassic, both of which were largely in the Southern Hemisphere.

## 4. Discussion

### 4.1. Temnospondyl trophic ecomorphology and evolution

Our functional-morphological study shows that the large diversity of temnospondyls in the Early Triassic spanned all feeding modes seen before and after, suggesting no selectivity across the PTME for a particular trophic ecology (figure 3). Further, the fact that fossils occur in a full range of sedimentary facies, from terrestrial and freshwater (rivers, lakes) to marine, suggests no habitat specialization.

Most Triassic capitosaurids and trematosaurids were predators, with a suite of adaptations for an aquatic mode of life, including relatively reduced limbs and strong lateral line sulci in the skull [88]. Overall cranial morphospace occupation is heavily skewed to the large parabolic morphotype, found in a range of temnospondyl groups including capitosaurs, basal stereospondyls and post-Induan trematosaurs (figure 3a). This low and generally parabolic skull morphology is somewhat similar to the crania of modern giant salamanders, such as *Andrias davidianus*, with similarities in cranial suture and mandibular morphology [85]. This large salamander has been proposed [89] as a plausible model for temnospondyl feeding function. The modern giant salamander feeds using asymmetrical suction strikes on its prey through unilateral jaw and hyobranchial movements, suggesting that Mesozoic temnospondyls might have shared a similar generalist trophic ecology (figure 1a). Our results corroborate recent work [25] that identified feeding guilds among temnospondyls based on landmarking analysis. They also found that large parabolic skulls indicate generalist taxa, by comparison with robust morph phytosaurs and extant crocodilians in their dataset. However, they suggest that *Metoposaurus* differed from large capitosaurs by adopting ambush feeding strategies, whereas we are unable to distinguish significant differences in metoposaur and capitosaur ecomorphology, suggesting they occupied a similar feeding guild, as confirmed by bone histological analyses [2,90].

Longirostrine forms are less common in the Mesozoic, appearing briefly in the Early Triassic and then disappearing in the Anisian (figure 4a). They are notably also present in marine environments, in contrast to the parabolic forms, which are restricted to fresh waters [12,91]. The longirostrine morphology is reminiscent of modern gharial crocodiles (Gavialidae) and gracile morph phytosaurs, suggesting a specialized piscivorous diet [25]. We confirm this similarity of skull shape and feeding mode (fast but weak bites) for *Aphaneramma,* which they identify in this feeding guild, but also for *Wantzosaurus*, which they identify as a feeding generalist, with which we agree. Reconstructed marine food webs suggest that longirostrine temnospondyls were largely mesopredators [12], but in freshwater ecosystems, they occupied higher trophic levels. The disappearance of longirostrine temnospondyls at the beginning of the Middle Triassic (figure 4a) coincides with the emergence of archosauromorphs like proterochampsids and later, phytosaurs [92–94]. This could suggest that the former were outcompeted by the latter, or that archosauromorphs evolved to fill these niches in tune with a combination of ecological factors such as climate change, resource availability or habitat loss [25,95]. This cannot currently be resolved.

Static morphospace occupation through the Triassic (figure 4a) appears to have stemmed from a stasis model of trait evolution (figure 5b). Evolutionary stasis can be described as the long-term stability of species morphology in an evolving lineage [96]. An exceptional example of Triassic temnospondyl stasis is *Gerrothorax pulcherrimus* [97]*,* an aquatic, suction-feeding ambush predator that inhabited river and lake bottoms for 35 Myr from Ladinian to Rhaetian (figure 4a). Its broad-skulled, benthic adaptation became standard for temnospondyls following the Early Triassic, with multiple clades exhibiting this morphology. High phenotypic plasticity could explain the apparent lack of morphological change as it can increase the probability of population persistence without substantial skeletal change [98].

### 4.2. Temnospondyls as PTME survivors

It has long been recognized that temnospondyl diversity was much higher after the PTME than before [6,7,99–102]. Shishkin [100] argued that the Induan to mid-Olenekian Triassic peak represented a time when temnospondyls were responding to crisis conditions and showed some of the characteristics of disaster taxa. He argued that loss of normal ecological interactions through fragmentation of ecosystems was reflected in cosmopolitanism of species, short-lived taxa (high turnover) and high morphological disparity. Certainly, most of the earliest Triassic temnospondyls show features associated with ecological success in times of crisis, namely, a large geographical distribution, small size and generalist diets. Modesto [103] likewise attributed the richness in diversity and disparity of Early Triassic temnospondyls to environmental stress, as exemplified especially by the Panchet and, to some extent, Knocklofty temnospondyl, assemblages from India and Australia, respectively [8,13].

Additional survival factors of the latest Permian temnospondyls include their ectothermy, the possible ability to burrow, and their semi to fully aquatic ecology [104,105]. Bone histology suggests that Permian temnospondyls were adaptively diverse, exhibiting a wider spectrum of preferred habitats prior to the PTME than after, when most Triassic forms were secondarily adapted to aquatic habitats [2,90], highlighting how aquatic environments may have been refugia across the PTME [13]. Many Permian temnospondyls were terrestrial predators, not limited to aquatic habitats. Large water bodies are generally cooler than the surrounding land meaning aquatic habitats could have acted as shelter against hothouse conditions [18].

There is only limited evidence of the Lilliput effect among earliest Triassic temnospondyls (figure 2). Many of the temnospondyls from Early Triassic formations in Australia (Knocklofty, Rewan, Arcadia) are small and these have been called dwarfs, corresponding to a time of halving of size that lasted through the Induan and early Olenekian. But size recovered to pre-extinction distributions 2 Myr after the PTME, in the early Olenekian, at least in South Africa [3]. Other earliest Triassic temnospondyls in Russia and elsewhere, however, retained typical body sizes seen before the PTB, suggesting the presence of sufficient prey to sustain medium-large temnospondyls in fluvial ecosystems. When considered separately, both trematosaurs and capitosaurs apparently increased in size through the earliest Triassic (figure 2).

### 4.3. Early Triassic diversity peak or sampling bias?

The Early Triassic peak in temnospondyl diversity and disparity could either be real or an artefact of over-sampling (e.g. one or more Lagerstätten; unusually high preservation of appropriate facies; over-representation of certain palaeolatitudes) or over-study (e.g. concentration of fossils in classic sites; monographic peak owing to the intense work by one or more researchers). Indeed, this peak is so high that Ruta & Benton [7] termed it the ‘Induan outlier’; they ran a variety of numerical analysis, checking the dating of formations and the impact of different tree topologies and taxonomic levels, but the peak occurred in every analysis suggesting at least that it is not a statistical artefact.

Evidence is weak that the Induan peak is an artefact of biased study. Induan-aged temnospondyls are reported from South Africa, Madagascar, Pakistan, India, Australia, Antarctica, Brazil, Uruguay, Greenland and Russia, but are rare in Germany [106] and absent in North America. This is contrary to normal expectations of over-study which would predict highest taxon counts in Europe and North America, where more researchers have been working for longer than in other continents. Further, the Induan and Olenekian temnospondyls from these regions were largely named in single publications and there are few monographs that present numerous new taxa and could be claimed to have artificially inflated numbers of taxa. In their exploration of possible worker bias in the study of basal tetrapods from the late Palaeozoic and early Mesozoic, Bernard *et al*. [107] found no correlation between the numbers of publications and reported taxic diversity of temnospondyls and relatives. They also found no evidence for bias by differential study of fossils from different time intervals, so the Early Triassic has not been ‘over-studied’ in comparison to the Late Permian or Middle Triassic.

Further, evidence of a geographic or facies bias could not be identified. While no Early Triassic temnospondyl-bearing formations are formally recognized Lagerstätten, localities such as Czatkowice show exceptional preservation approaching Lagerstätte quality. The sedimentological facies represented are terrestrial red beds and include a variety of river- and lake-deposits, but generally not aeolian [108,109], but they are restricted to temperate and polar palaeolatitudes of the Triassic. Further, the fossils come from a broad geographic range around the northern and southern temperate zones. So, we cannot rule out preservational bias, but neither can we find evidence for such a bias.

### 4.4. The Early Triassic ‘Tropical Dead Zone’ and recovery from the PTME

The apparent absence of diverse fossil faunas and floras from the tropics through much of the Early Triassic was noted by Sun *et al.* [15], who argued that these absences reflected a real phenomenon, the ‘Tropical Dead Zone’ (TDZ). They suggested that a broad palaeolatitudinal belt around the tropics was too hot for life to survive on land or in shallow seas. Oxygen isotopic studies of marine and nonmarine sediments show up to four peaks of elevated temperature through the first 6 Myr of the Triassic (Payne *et al*. 1994), or even prolonged high temperatures, 10−15°C higher than normal, through the Early Triassic [14].

On land, these extreme temperatures would have driven life towards higher latitudes; in the oceans, organisms could perhaps have survived at mid water depths, above the anoxic seabed, but mostly probably migrated north and south to cooler waters [16]. Indeed, skeletal and footprint evidence suggests a distinct shift poleward by 10−15^o^ among tetrapods from latest Permian to Early Triassic [17], supporting a general migration to escape superhot equatorial climates. Nonetheless, the TDZ does not appear to have been permanent through the Early Triassic because tetrapod fossils are reported from the western United States, southern Europe, north Africa and south China [15,17]. Perhaps cooler equatorial conditions prevailed between outbursts of volcanic eruptions and global warming.

Our biogeographic reconstructions (figure 6) [58] (electronic supplementary material, figures S2) suggest that Permian stem-stereospondyl lineages diversified primarily in Gondwana, but latest Permian taxa appear distributed across all biogeographic zones, suggesting some complexity of the TDZ. During the Early Triassic, the equatorial region was permeable at times (figure 6a,b). This permeability may support the suggestion [13] that the TDZ varied in latitudinal extent through the Early Triassic, being sometimes wide, sometimes narrow, depending on levels of hyperthermal activity and varying global temperature [23]. Further, waterways through this TDZ may have been sufficiently shielded from adverse climatic conditions to support limited terrestrial faunas that were tied to aquatic resources [11,13]. More study of the Early Triassic TDZ is needed to determine how it waxed and waned through its 6 Myr existence.

Temnospondyl temperature tolerances are thought to have been similar to those of contemporary amphibians and fish, giving them physiological limitations that should have forced temnospondyls into higher latitudes [12,18,106]. As noted [18], their aquatic lifestyles combined with generalist trophic ecologies might have helped temnospondyls to survive at lower latitudes during the hothouse conditions, in areas where fully terrestrial tetrapods could not. Our phylogenetic estimations highlight these potential equatorial pockets of temnospondyl survival, with many Early Triassic node states suggesting diversification in equatorial regions (figure 6c) [58] (electronic supplementary material, figures S2). The DEC model, which had stronger support (loglikelihood: −153, AIC: 310) compared to the DIVALIKE model (loglikelihood: −169.1, AIC: 342.22), shows slightly stronger dispersal in equatorial regions through the Permian–Triassic transition and Early Triassic. Note, however, that our reconstruction uses only three geographic belts, so cannot test for more subtle patterns. Despite some presence in equatorial regions, temnospondyls generally diversified in the more temperate/polar regions of both supercontinents (figure 6c) [61] (electronic supplementary material, figures S2).

The traditional assumption about ecosystem construction [19] is that following the PTME there would have been bottom-up recovery, namely that primary producers re-established themselves first, then primary consumers, followed by secondary consumers and higher levels of top carnivores last. However, study of earliest Triassic marine ecosystems suggests something quite different, with the emergence of top-level predatory tetrapods and sharks very early in the Triassic [12,110]. Brayard *et al*. [110] suggested that the apparent disorder in recovery of ecological levels is not so surprising because numerous higher-level predators belonged to groups that survived the PTME, perhaps because they were generalists without specialized diets. Therefore, new clades of predatory tetrapods emerged rapidly in the Early Triassic, including the marine trematosauroids, but also ichthyosauromorphs and eosauropterygians [111]. The differing ecomorphologies of marine and freshwater temnospondyls and their prevalence in the earliest Triassic illustrate that temnospondyls were a major part of this diversification of predators [111] and highlights how their aquatic lifestyles supported their Early Triassic success as well as their survival through the PTME.

### 4.5. Links between temnospondyl biogeography and patterns of diversification

Environmental conditions changed substantially through the Triassic, and this might have affected temnospondyl distributions. The rapid shifts in climate might have driven substantial changes in vegetation and faunal extinctions and radiations [17,18], and temnospondyls would probably have experienced changes in their selection pressures from these external factors.

The difference between the temnospondyls of Laurasia and Gondwana (*p* < 0.01) could indicate that barriers between the two landmasses supported divergent specialization across niches unique to each continent. Gondwanan taxa were at their most morphologically disparate during the Early Triassic (figure 6c), featuring longirostrine and broad-snouted aquatic forms, and even more terrestrial forms such as the lydekkerinids [2,3,112] (figure 4a). While it may be an artefact of preservation or sampling bias, our data could suggest that the southern continents supported a greater variety of temnospondyl body plans and by extension functional ecospace, during the Early and Middle Triassic.

Following the Early Triassic, temnospondyl morphological diversity became more globally homogenous, although there were some geographic differences. It appears that equatorial and Gondwanan temnospondyls enjoyed greater ecological freedom to diversify across a wider variety of aquatic niches in the Middle Triassic [61] (electronic supplementary material, figures S2). Capitosaurids grew to large sizes and became apex predators in Laurasian waterways during the Middle Triassic, whereas Gondwanan temnospondyls appear to have remained much smaller, likely occupying mesopredatory niches. Some of the later capitosaurs such as the tropics-based eocyclotosaurids had proportionally longer skulls that converged on the morphology of longirostrine trematosaurs from the Early Triassic (figures 4a and 6c).

### 4.6. Later temnospondyl evolution

The Carnian marked another interval of profound climatic and faunal change in the Triassic, primarily through the Carnian Pluvial Episode (CPE; 234−232 Ma), an interval of high humidity and recurrent heavy rainfall [113]. While temnospondyl disparity fell from a peak in the Early Triassic, it stabilized in the Carnian through the short-term success of the metoposaurids, cyclotosaurids and plagiosaurids (figure 4). This success was geographically widespread (figure 6) [61] (electronic supplementary material, figures S2) as metoposaurs radiated across equatorial and southern regions, whereas cyclotosaurids and plagiosaurids diversified across northerly European tropics [114,115]. Following the CPE, metoposaurid diversity and distribution plummeted (figure 4a,b), highlighting the sensitivity of this clade to environmental conditions [116], when the stable temperatures, regular precipitation, and low seasonal variation in their water bodies broke down [13,117].

After the Carnian, temnospondyl spatial distribution became extremely fragmented and incomplete: Metoposauroidea are the most diverse, Capitosauria were largely extinct, and Chigutisauridae began to diversify. Derived chigutisaurids survived past the CPE, with fossils from Argentina and India [42,118], raising questions about their direction of travel between east and west Gondwana. Metoposauroids dispersed between Europe and North America (figure 4a) [61] (electronic supplementary material, figures S2).

Indeed, temnospondyls became much rarer following the CPE, in comparison to archosauromorph and synapsid tetrapods [117]. This decline coincided with increasing aridity across much of Pangea in the Norian [21]. Palaeosols indicate strong seasonality, and more localized precipitation in coastal areas, while areas in low to mid latitudes underwent aridification [119]. The shift in climate may have driven the gradual extinction of the remaining major temnospondyl lineages at the end of the Rhaetian (201.3 Ma). Only one temnospondyl lineage survived the Triassic–Jurassic extinction event, the Chigutisauridae, some of which retained relatively large sizes (figure 6c). We confirm that temnospondyls become increasingly rare in the Late Triassic (figure 4a), though evidence that this decline was driven by competition with archosauromorphs [25] is not clear. Changing climates might well have been the main driver. Late Triassic lake environments appear to have been large enough to support an array of semi-aquatic tetrapods, as illustrated by the coexistence of cyclotosaurids, metoposaurids, plagiosaurids and phytosaur archosauromorphs, with apparent ecological partitioning allowing a remarkable diversity of carnivores, with metoposaurids as mesopredators, and cyclotosaurids or phtyosaurs likely being the apex predators, depending on the locality [120]. Chigutisaurids such as *Siderops,* which shares parabolic skull morphology with capitosaurs, are found in Toarcian deposits (Early Jurassic), and continued as large generalists until the Cretaceous (e.g. *Koolasuchus cleelandi*), perhaps restricted to Gondwana.

## 5. Conclusion

We have explored reasons for the diversification and success of temnospondyls in the Early Triassic. Patterns of morphological evolution through the PTME show little change in their morphology through the mass extinction event, with new forms instead emerging shortly afterwards. Body size in some clades diminished for 1−2 Myr, but any such Lilliput effect was limited by clade or geographically, and pre-extinction body sizes were quickly achieved again after the crisis. We cannot point to any evidence for a preponderance of any particular feeding mode that might reflect some ecological shift to deal with the stringent, hot and arid conditions of the earliest Triassic. Indeed, their burst of disparity in the Olenekian was brief and they largely reverted to a core ecomorphology through the remainder of the early Mesozoic. Strong ecomorphological conservatism as highlighted by support for the OU and stasis models of evolution suggests that temnospondyl success lay in their generalist feeding ecology, enabling them to feed on a wide variety of prey despite the array of environmental changes happening all around them through the Triassic. Broader consideration of Triassic ecosystems perhaps also indicates that the freshwater ecosystems they preferred, provided temnospondyls with a relatively stable array of food resources, allowing them to thrive while strictly terrestrial predators sufficed with meagre, unstable resource availability on land [121].

Temnospondyl palaeogeographic distributions show that they exhibited a greater presence in equatorial regions than expected, raising questions about the strength of dispersal barriers and apparent lifelessness across the TDZ. It may be that their generalist feeding ecology combined with their ability to exploit aquatic resources facilitated temnospondyl survival in these hot, arid, largely inhospitable areas. Further study should reveal whether equatorial freshwater environments were refugia and/or dispersal corridors into and across the tropics. Nonetheless, we confirm a general preference among temnospondyls for higher latitude habitation until conditions in equatorial climates ameliorated from the Middle Triassic onwards. We do identify a significant difference of ecomorphologies between Gondwana and Laurasia. Gondwana appears to have been the last refugium for these disaster taxa, but sample size and preservation biases may impact these results.

Temnospondyl disparity decreased through the Middle–Late Triassic. During the Carnian, the CPE seemed to facilitate the stabilization and diversification of some derived trematosauroid lineages, notably Chigutisauridae and Metoposauroidea, perhaps linked to the spread of aquatic environments. By this time, the large capitosaurs had largely died out (only the cyclotosaurids remained) and temnospondyls were far rarer in Upper Triassic deposits. Our morphospace analyses lack sufficient resolution to split the large capitosaur and metoposauroid cluster and we were unable to find distinct feeding guilds, but our findings largely corroborate recent skull morphology analyses, and highlight strong selection for the parabolic morphology in Mesozoic temnospondyls.

## Data Availability

Supplementary material is available online [122].
